# The quality and diagnostic value of open narratives in verbal autopsy: a mixed-methods analysis of partnered interviews from Malawi

**DOI:** 10.1186/s12874-016-0115-5

**Published:** 2016-02-01

**Authors:** C. King, C. Zamawe, M. Banda, N. Bar-Zeev, J. Beard, J. Bird, A. Costello, P. Kazembe, D. Osrin, E. Fottrell, for the VacSurv Consortium

**Affiliations:** Institute for Global Health, University College London, 3rd Floor, 30 Guilford Street, London, WC1N 1EH UK; Parent and Child Health Initiative, Lilongwe, Malawi; MaiMwana Project, Mchinji, Malawi; Malawi-Liverpool-Wellcome Trust Clinical Research Programme, College of Medicine, University of Malawi, Blantyre, Malawi; Institute of Infection & Global Health, University of Liverpool, Liverpool, UK; London School of Hygiene and Tropical Medicine, London, UK; Department of Computer Science, City University London, London, UK; Baylor College of Medicine Children’s Foundation, Lilongwe, Malawi

**Keywords:** Verbal autopsy, Open narrative, Closed questions, Bias, Sub-Saharan Africa

## Abstract

**Background:**

Verbal autopsy (VA), the process of interviewing a deceased’s family or caregiver about signs and symptoms leading up to death, employs tools that ask a series of closed questions and can include an open narrative where respondents give an unprompted account of events preceding death. The extent to which an individual interviewer, who generally does not interpret the data, affects the quality of this data, and therefore the assigned cause of death, is poorly documented. We aimed to examine inter-interviewer reliability of open narrative and closed question data gathered during VA interviews.

**Methods:**

During the introduction of VA data collection, as part of a larger study in Mchinji district, Malawi, we conducted partner interviews whereby two interviewers independently recorded open narrative and closed questions during the same interview. Closed questions were collected using a smartphone application (mobile-InterVA) and open narratives using pen and paper. We used mixed methods of analysis to evaluate the differences between recorded responses to open narratives and closed questions, causes of death assigned, and additional information gathered by open narrative.

**Results:**

Eighteen partner interviews were conducted, with complete data for 11 pairs. Comparing closed questions between interviewers, the median number of differences was 1 (IQR: 0.5–3.5) of an average 65 answered; mean inter-interviewer concordance was 92 % (IQR: 92–99 %). Discrepancies in open narratives were summarized in five categories: demographics, history and care-seeking, diagnoses and symptoms, treatment and cultural. Most discrepancies were seen in the reporting of diagnoses and symptoms (e.g., malaria diagnosis); only one pair demonstrated no clear differences. The average number of clinical symptoms reported was 9 in open narratives and 20 in the closed questions. Open narratives contained additional information on health seeking and social issues surrounding deaths, which closed questions did not gather.

**Conclusions:**

The information gleaned during open narratives was subject to inter-interviewer variability and contained a limited number of symptom indicators, suggesting that their use for assigning cause of death is questionable. However, they contained rich information on care-seeking, healthcare provision and social factors in the lead-up to death, which may be a valuable source of information for promoting accountable health services.

## Background

In the absence of universal vital registration systems, and with large numbers of deaths occurring without medical attendance, verbal autopsy (VA) is widely used to identify cause-specific mortality patterns in low- and middle-income settings [1]. VA is the process of interviewing close contacts of the deceased to identify probable causes of death, typically comprising predetermined closed questions about specific signs and symptoms, e.g., ‘in the illness preceding death, did the deceased have a fever?’.

This closed interview is often accompanied by an open narrative section in which the respondent’s account of events and circumstances leading to death is recorded by the interviewer. This details the sequence of events recalled and reported by the respondent without prompting, and can include information on social factors such as beliefs about the aetiology of the illness and health seeking behaviours [2–4]. Whether this narrative is recorded verbatim or transcribed as a summary of key events varies with intended use of the data and local standard operating procedures. Differing interviewer skill levels may introduce bias if interviewers consciously or otherwise record narratives that fit neatly into preconceived disease descriptions (e.g., fever corresponding to malaria) [5, 6]. Field experience from South Africa suggested that the open narrative is the most time consuming and emotionally upsetting part of the VA process for both interviewers and respondents (unpublished focus group discussion data – EF and J Bird).

In 2006, 18 different VA tools were in use across demographic surveillance sites in Africa and Asia, some relying heavily on open narratives while others used only closed questions [7, 8]. Recent efforts by the World Health Organization (WHO) attempt to limit this inconsistency with standardized VA tools, but the open narrative section remains optional and its usage is likely to be variable [1]. As with any questionnaire method, VA data capture tools must be designed with data analysis methods in mind. Traditionally, physician-coded VA (PCVA) methods, in which data are reviewed by physicians to identify likely causes of death, have been commonly used. The extent to which physicians base their cause of death ascertainment on data from closed questions and/or the open narrative is unknown, but likely varies between individuals and causes of death. Previous work has shown that the amount of information available to the physician and their knowledge of the local epidemiology and disease profiles affect their cause assignment [9], and that agreement between physicians is related to the age and sex of the deceased [10]. To overcome potential bias, reduce cost, and increase timeliness and reliability, Computer Coded VA (CCVA) methods are now widely used and recommended for large-scale VA studies [11]. CCVA methods apply statistical reasoning to calculate the most likely causes of death given the symptoms reported. Although it is possible to derive answers to closed questions from open narratives, including these data in CCVA methods has not demonstrated substantial differences to causes of death assigned [12–14] and current CCVA methods typically use closed questions only.

A key consideration in the use of open narrative text for either CCVA or PCVA approaches is the completeness and reliability of the unstructured information. Several factors are known to influence the quality of VA data, such as respondent recall and characteristics of the deceased and the respondent (e.g. age at death) [11, 15]. However the extent to which an individual interviewer, who generally does not interpret the data, affects the cause of death outcome is poorly documented. The aim of this paper is to examine inter-interviewer reliability of open narrative and closed question data gathered during VA interviews, and assesses the diagnostic influence of interviewers on final cause of death determination using PCVA and CCVA methods.

## Method

We conducted a mixed methods study to investigate the reliability of data capture in verbal autopsies. This was done by comparing concurrently collected and merged open narrative and closed questions from partnered verbal autopsy interviews for deaths in children under five in Mchinji District, central region, Malawi. Data were collected between March and April 2013 as part of a larger paediatric vaccine effectiveness cohort study in which we are conducting VAs for all deaths in children under 5 in Mchinji district [16]. Malawi is a low-income country and has an under-five mortality rate of 71/1,000 livebirths, with significant reductions in this rate since 2000 [17]. Mchinji district has a population of approximately 465,000, of whom 85 % live in rural communities and are subsistence farmers [18]. Currently Malawi does not have a comprehensive vital registration system, and only deaths which occur within hospitals are recorded.

### Ethical approval

Ethical approval for the work was granted by the National Health Sciences Research Committee of Malawi (ref: #837). Senior fieldworkers read a study information sheet and explained the consent process, and verbal consent was sought from respondents prior to interview, and their consent was recorded in the electronic form.

### Data collection

Within the prospective community key event surveillance system, we conducted VAs for all under-five deaths [16]. Deaths are identified and reported monthly to field supervisors by trained village-level volunteer informants who cover a catchment area of approximately 80 households. The data are checked and then submitted to the central office for processing and cleaning. Under-five deaths were listed from this system, and interviewers approached the families of the deceased at their homes to conduct the interviews.

Interviews were conducted by 8 senior fieldworkers, all of whom were well-versed in local cultural norms and had over 5 years experience in conducting VAs during prior research studies (which used both closed questions and open narratives). They received 1 week’s refresher training, which covered definitions of the closed questions and translation, handling the emotion of the interviews sensitively, study protocol, use of smartphones for data collection, and mock interviews. The interviewers, all of whom were competent English speakers, contributed to the translation of the closed questions into Chichewa, ensuring that there was a common understanding of the questions and their meaning in the local language and cultural context. All interviews were conducted in Chichewa.

VA closed question information was collected using the mobile-InterVA (MIVA) application running on Android smartphones [19]. MIVA is based on the standardized WHO 2012 VA questions [20], and in-built skip patterns based on age and symptoms reported allows 44 – 104 neonatal questions and 34 – 101 infant and child questions [21]. This tool is designed to be compatible with the CCVA tool ‘InterVA’ (www.interva.net) for assigning cause of death, which uses a Bayesian approach to assign a weighted cause of death based on positive responses to the closed questions. Unstructured open narratives were elicited before the closed questions and fieldworkers recorded information in either Chichewa or English, depending on fluency and personal preference. The two sources of information were linked through a scanned unique ID barcode and interviewer’s ID number. Open narratives were recorded on paper and then entered (after translation into English, when necessary) into a Microsoft Access database.

### Partner interviews

**Fig. 1:**
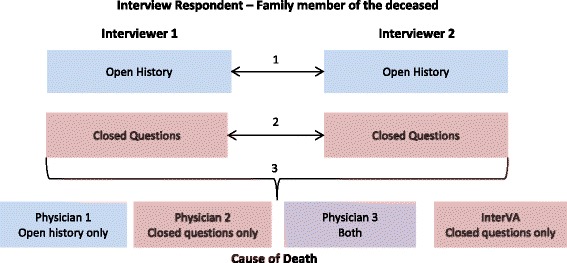
Schematic of data collection, interpretation and analyses presented. 1: comparison of open narrative data between interviewers; 2: comparison of closed question data between interviewers; 3: comparison of causes of death for the same child from information collected between interviewers

As the initial phase of field roll-out, fieldworkers conducted VAs in pairs (i.e. a partner interview - Fig. 1). *Partner interview* refers to the single event with two senior fieldworkers (‘interviewers’) concurrently recording closed question and open narrative information from the caregiver of the deceased. In the partner interviews, the fieldworker responsible for the geographical cluster in which the interview was taking place asked the questions, and both fieldworkers recorded both the open narrative and closed questions concurrently, resulting in two *interview records* (data collected by a single interviewer within the partner interview). A supervisor was present to assist with technical issues with the smartphones. The data collected during these partner interviews are the basis for the current analysis, giving two versions of the open narrative and closed questions for every interview conducted.

### Quantitative analysis

We calculated the inter-interviewer percentage agreement for the closed questions in each pair of interview records, by dividing the number of concordant answers by the total number of questions asked. We calculated the Krippendorff alpha as a statistical measure of reliability, with values closer to 1.00 indicating high reliability [22, 23]. This was done using Stata SE11 [24], and the analysis gives the inter-interviewer reliability for the closed questions. *Inter-interviewer* refers to analyses looking at the differences in data captured by the two interviewers during the same interview event.

We compared the causes of death assigned between the interview records. Cause of death was determined for complete pairs of partner interviews by three independent physician reviewers and InterVA (as an example CCVA method). One physician was given the closed questions only, one the open narratives and the closed questions, the third the open narratives only, and InterVA used closed question data only – in all cases they were given both sets of data from each interview. All reviewers had over 20 years’ experience in paediatrics or neonatology in low-income countries and had reviewed VAs previously. They were asked to assign cause(s) of death based on the data they were presented with and were permitted to assign multiple causes of death for each case. InterVA can assign up to three causes of death, and gives each cause a probability weighting. Reviewers were assigned data sources randomly and were blinded to the research questions, to each other, and to the partnered nature of the interviews. Causes were categorized using the WHO 2012 ICD-10 list of causes for verbal autopsy [20]. If any cause of death assigned was the same between the interview records (irrespective of number of causes assigned), it was considered to be in agreement and the percentage agreement was calculated. This analysis investigates the consistency in information provided in the closed questions and open narrative and how this influences cause of death, both for PCVA and CCVA methods.

### Qualitative analysis

We investigated the reliability in information recorded in the open narratives between interview records, with differences in the open narratives coded deductively (CK) and checked by a second reviewer (CZ). These differences were grouped and summarized under emergent themes, and major and minor differences coded. We defined *a priori* a major difference as directly contradictory information and missing or additional events, symptoms or diagnoses (e.g. a hospital admission, or HIV status); a minor difference is a variation of the same event, symptom or diagnosis (e.g. unspecified treatment versus an antibiotic drip).

To investigate potential added information that open narratives may provide, we conducted a qualitative framework analysis of the open narrative data. We pre-defined five broad themes, the first three based on the principle of three delays described by Thaddeus and Maine (1994): delay 1 (decision to seek care); delay 2 (getting to healthcare); delay 3 (provision of adequate care) [25, 26]; perceptions of the circumstances which caused the death; and medical symptoms. Themes were agreed in discussion by CK and EF before analysis, and were based on topics thought to be lacking from the closed questions; medical symptoms was included to capture any information not currently asked in the WHO 2012 VA tool. Sub-themes were defined in the framework as they emerged from the coding matrix.

## Results

A total of 18 deaths were followed up for a partnered VA. All fieldworkers conducted at least two interviews, with 17 partner interviews having complete closed question data, 12 complete open narrative pairs and 11 with matched complete data. Reasons for incomplete data included technical issues in saving closed questions (*n* = 1), incorrect recording of interview ID information on open narrative forms (*n* = 3), and loss of paper forms (*n* = 3). Interviews included 8 neonatal, 3 post-neonatal infant and 7 child deaths, with one interview reporting a discrepancy in death type (1–5 years vs. 6–14 year old).

### Closed question reliability

On average 66 (IQR: 64 - 69) questions were asked in neonatal VAs, 70 (IQR: 59 - 82) for infants and 70 (IQR: 68 - 74) for child deaths, of which an average of 17, 19 and 20 were ‘yes’ responses (the information used by InterVA to calculate cause of death). The median number of differences between interview records was 1 (IQR: 0.5 – 3.5), with four interviews having no differences; the average inter-interviewer agreement was 92 % (Table 1). The interview with discrepant age categories had a lower agreement of 81 %; this was not unexpected as some of the questions displayed would have been different. The Krippendorff alpha was 0.90 when considering agreement in ‘yes’ responses only and 0.88 when comparing all responses (i.e., yes, no and don’t know), indicating good inter-interviewer reliability.

**Table 1:** Inter-interviewer reliability of closed questions

	Agreement Mean (IQR)	Reliability (Krippendorff’s alpha)
All responses	92 % (92–99)	0.88
‘Yes’ responses	96 % (95–99)	0.90

*WHO* World Health Organization, *VA* verbal autopsy, *IQR* inter-quartile range

### Open narrative reliability

The average number of signs and symptoms that corresponded to WHO 2012 VA questions in the open narrative texts was 15 (range: 4 – 22), with an average of 9 (range: 3 – 14) symptoms reported as present in the lead up to death. Differences were summarized in five categories: demographics; social/cultural; history and care-seeking; diagnoses and symptoms; treatment. One interview demonstrated no differences in the narratives, with an average of 2 differences per pair and most discrepancies found in diagnoses and symptoms (Table 2).

**Table 2:** Summary of discrepancies between partner interview open narratives

Theme	Pairs with discrepancies (%)	Mean discrepancies^a^	Example discrepancy (major/minor)
Demographics	5 (42 %)	1.9	Exact date of death vs. month of death (minor)
Social/cultural	3 (25 %)	1.0	“That night we heard a sound like people are pounding maize…” vs. no mention (major)
History and care-seeking	6 (50 %)	1.2	“I took the child to [referral hospital] where [he] was admitted for 4 days” vs. no admission (major)
Diagnoses and symptoms	10 (83 %)	2.8	“The child had malaria” vs. no mention (major)
Treatment	8 (67 %)	1.1	“…put on a drip of blood and a drip of water” vs. “we were again given treatment” (minor)

^a^Using the number of pairs with discrepancies as the denominator in calculating the mean

#### Demographics

Differences in demographics (e.g. age at death) generally consisted of one interviewer recording specific dates and precise ages, while the other was vague or did not report at all. Gender was not specifically mentioned in any narrative and pronouns changed throughout the text, reflecting the lack of gender specific pronouns in Chichewa.

#### Social/cultural

Three interviews had differences reported in the cultural and social context of the death (i.e. non-medical). For example, one interview record implied witchcraft: *“that night we heard a sound like people are pounding maize, this happened for at least 20 min … while she was sleeping like that, her father heard the same night [the] sound of people pounding”* (interviewer 2, child death). The other interview record did not include these details. Other differences related to the tone in which the narrative was written, sympathizing with the care given: “*they [healthcare worker] were regretting the failure to refer me*” (interviewer 5, neonatal death), compared to an accusatory tone regarding the care received: “*[the baby died] because the mother called the nurse but the nurse said she should still wait*” (interviewer 6, neonatal death).

#### History and care-seeking

Half of the partner interviews had differences in recording of the medical history and care seeking. This was generally around the frequency and location of care sought; e.g., “*I took the child to MDH [referral hospital] where [he] was admitted for 4 days*” (interviewer 3, infant death), while the other interview record did not mention an admission.

#### Diagnoses and symptoms

The differences in medical diagnoses and symptoms recorded ranged from minor to major (as defined in the Methods). Of note were examples in which “*the child had malaria*” (interviewer 3, infant death) or “*the child [was] HIV positive*” (interviewer 3, child death), appeared in one record but not the other. Major differences were observed in five and minor differences in ten of the interview pairs, with an average of 2.8 differences observed.

#### Treatment

Treatment information was different in three-quarters of pairs, such as, *“the baby was being given some 7 injections as medication for pneumonia and some panadol too”* (interviewer 5, child death), compared to no mention of either injections or pneumonia.

### Cause of death

Cause of death agreement is presented in Table 3. Inter-interviewer comparability of cause of death by physician review increased with closed questions, either in isolation (82 %) or in combination with open narratives (82 %), when compared to open narratives alone (55 %). Physician review had better inter-interviewer agreement than InterVA when assigning cause of death based on closed questions alone (82 % vs. 55 %). The main source of disagreement in causes assigned were between septicaemia and malaria, and inconsistencies in early neonatal causes of death (i.e., prematurity and birth asphyxia); deaths due to accidental and congenial malformation causes had good agreement.

**Table 3:** Cause of death, according to different data sources and interpretation method

	Physician review			InterVA
	Closed questions only	Open narrative only	Both	
Number of assigned causes *mean (range)*	1.6 (1–3)	1.1 (1–2)	2.3 (1–5)	1 (1)
Inter-interviewer comparison^a^ *no. concordant pair (%)*	9 (82 %)	6 (55 %)	9 (82 %)	6 (55 %)

For all review methods 11 interview pairs (22 interview records) were analysed

^a^Inter-interviewer comparison represents the number interviews in which the same cause of death was assigned for any cause (not all causes)

### Additional information from open narratives

#### Delay 1

All pre-defined themes were found throughout the open narratives. Half the records mentioned a delay in deciding to seek healthcare, which were further categorized into not considering it serious and the time taken to make the choice. One respondent said that they “*didn’t take it as a serious health problem*” (interviewer 5, child death), and therefore did not seek care at that time. Six of the narratives said that the child’s illness (including fevers, vomiting and cough) lasted for 2–3 days before they decided to seek any care.

#### Delay 2

Delay in getting to healthcare services was mentioned in four narratives, with one mention of a delay in referral and the remaining a delay in transport availability. These delays were related to a lack of ambulances and limited transport options: “*we were referred to MDH [referral hospital] but there was no transport. They said we should ride a bicycle to [the town]*” (interviewer 4, neonatal death) a journey of over 30 km.

#### Delay 3

Delay in receiving appropriate care was reported in almost half the open narratives. We defined four sub-categories: lack of equipment or medication, lack of staff, lack of assistance from staff, and poor delivery of care. The lack of staff and lack of assistance highlighted malfunctions in health service delivery; for example, “*she was taken back to the hospital where we did not find any doctor… she delivered while there were not any medical personnel*” (interviewer 4, neonatal death), or “*some health workers were there but they denied to assist me”* (interviewer 3, neonatal death). There were narratives which were coded under multiple sub-themes, such as an example in which health centre staff failed to find forceps, and “*the doctors inserted their hands into me to pull the baby out… this did not help at all*” (interviewer 5, neonatal death).

#### Perceptions

Three sub-themes were categorized under perceptions of the cause of death: traditional beliefs, respondent understanding of cause of death, and overall well-being. Traditional beliefs, in addition to the witchcraft example described above, included seeking traditional medicine: “*it’s part of our culture to use the African medicine when you are pregnant*” (interviewer 3, neonatal death). Cause of death was reported in both non-medical (e.g., “*if we were given transport in good time I think we would have saved the life of the baby*” – interviewer 4, neonatal death) and medical senses (e.g., “*she died because of lack of blood [anaemia]*” – interviewer 8, infant death), allowing respondents to give their interpretation of events. Perceptions of medical causes were based on cultural knowledge in some cases, e.g., “*the child had fever and vomiting which shows that she had malaria*” (interviewer 3, child death). Perceptions of health in general also included positive aspects: “*his weight was not going down, it was something very boastful*” (interviewer 1, child death).

#### Medical symptoms

For medical symptoms, three sub-themes were defined: malnutrition, other specific terms, and family history (such as maternal epilepsy). Two pairs of interviews listed specific details relating to malnutrition, including referral to the inpatient district nutrition rehabilitation unit. Nearly half the narratives listed a specific “symptom” which would not have been fully captured by the closed questions, although the relevance of several is questionable. Examples include, “*she was trying to wake up on her own*” (interviewer 1, neonatal death) and “*she was failing to stretch her arms*” (interviewer 2, neonatal death).

## Discussion

We compared information from different components of VAs, collected simultaneously by two interviewers. Using this information we aimed to look at data reliability issues for open narratives and closed questions, as well as the potential for additional information gathered from the open narrative. We found that open narratives showed a high level of discrepancy between interviewers, especially in symptoms reported, and showed less agreement on cause of death by PCVA. However, these data contained further detailed information on cultural attitudes and health service delivery. In comparison with closed questions, open narratives showed more differences between interviewers, recorded fewer WHO 2012 VA indicators, and led to less consistent cause of death assignment from physician review.

### Reliability

The origin of inter-interviewer discrepancies in both closed questions and open narratives is important for understanding and improving data quality. We collected responses to closed questions using smartphones, and for all the fieldworkers this was their first experience doing so. It could be expected that some errors would be made as they familiarized themselves with the new software and hardware. However, we found little indication of difficulties in recording the closed questions accurately with a high inter-interviewer agreement.

The open narratives, however, had more discrepancies, mostly within treatment, diagnoses and symptoms (Table 2). Despite the interviewers being experienced, there was evidence of interpretation (or selective reporting) of respondent narratives of illness. This may come from personal biases based on pre-existing medical knowledge both from the interviewers and respondents, and the difficulty in expressing clinical details in the local language and context – although without recording the caregiver narratives we were unable to determine which narratives were more accurate. For example, in Chichewa the word for ‘fever’ also means ‘malaria’, so it is understandable that an interviewer might interpret the reporting of symptoms differently, depending on their understanding of the terms and surrounding circumstances. The interviewer’s beliefs and experiences might influence what they decide to be important information to record, with more scope for this to influence the content of an open narrative. While a study from Europe found that interviewer health beliefs did not influence the data recorded [27], this may not be applicable to a rural sub-Saharan African setting where traditional beliefs are common. This was apparent in the interview in which one interviewer reported on the role of witchcraft, while the other interviewer chose not to.

### Cause of death

The variation in causes of death assigned by physicians was not unexpected [28]. Variation in data quality and completeness between the data sources, with closed questions providing more consistent and complete data than open narratives, would lead to discrepancies. For the physician assigned causes, the more information they were provided with the number of causes which they assigned also increased. This could reflect more uncertainty in deciding cause of death when given more information – however, it did also lead to more agreement. Interestingly, the causes of death assigned using the CCVA approach with InterVA demonstrated poor consistency, and had the same agreement as using only open narratives and physician review (Table 3). This suggests that, while the closed question data were more reliable, CCVA methods may be more susceptible to minor inaccuracies than PCVA. This is not necessarily surprising as the routing of questions and analysis path for assigning weighted causes of death would be different based on different question responses.

### Additional information from open narratives

In spite of the fact that the open narratives performed poorly on quantitative measures of data quality, they may contain a wealth of social, cultural and programmatic information if they can be captured and interpreted consistently (e.g. delays in seeking and receiving appropriate care). This highlights possible areas for additional indicators to be considered for the WHO VA tool, as this is a tool which is continually evolving based on user feedback and advances in CCVA analysis methods [29]. One example might be intention to treat rather than receipt of treatment. The open narratives highlighted that, while healthcare was often sought, in nearly half of cases there were shortcomings in the care received according to the deceased family or caregivers. Currently, there are questions in the WHO 2012 VA tool (and 2014 tool [29]) about medications or treatments received. However, these questions are unable to make an important distinction between not needing the treatment and there being an intention to treat but insufficient resources to achieve treatment. The open narrative could be a rich data source for local health systems to use to target quality improvement, but its role in a standardised VA tool for scale-up in routine vital registration is questionable [30].

### Limitations

The number of records was small for the quantitative evaluations. It was designed as part of a supervised initial phase of field roll-out of the study protocol and quality assurance for mobile data collection prior to full field roll-out, and the number of interviews conducted was limited. Secondly, full partner data were not available for all the interviews conducted. Reasons for missing data were related to issues in implementation of the field exercise, and not related to the content of the interviews, so the study is unlikely to be biased by these missing data. The aim of this field test was to identify areas of misunderstanding and correct them, specifically with issues in mobile data collection. All interviewers had extensive prior experience of conducting VAs using paper forms and recording open narratives, and we would have expected data quality to be poorer in the electronically captured closed questions, which this descriptive analysis suggests were more robust.

## Conclusion

Our results suggest that there would be limited value in adding unstructured open narratives to VA materials in order to classify biomedical cause of death; the use of more structured narrative approaches (e.g., http://vatraining.vm-host.net/) could improve inter-interviewer reliability and replicating a partner interview comparison with more rigorous open narrative methods would be valuable. From a programmatic view, however, the added information on service delivery and community perceptions of health leading up to death might be important for evaluating interventions and stimulating accountability for service provision. The influence of the cultural context, such as localized beliefs about the aetiology of illnesses, may have different impacts on open narrative reliability in different contexts, and is something worth investigating across settings. Further research is needed into the effect of removing open narratives on the rapport between interviewers and respondents, the subsequent quality of closed question data, and the potential for including additional closed questions relating to the three delays.

### Availability of supporting data

Data used in this manuscript is available on request from the corresponding author (c.king@ucl.ac.uk), following the signing of a data sharing agreement.

## Abbreviations

PCVA: physician coded verbal autopsy
CCVA: computer coded verbal autopsy
VA: verbal autopsy
